# Machine Learning‐Based Development of Gadolinium Binding Peptides

**DOI:** 10.1002/nbm.70381

**Published:** 2026-09-11

**Authors:** Nir A. Dayan, Makayla Long, Nicolas Scalzitti, Iliya Miralavy, Daniel Holmes, Mark Kocherovsky, Wolfgang Banzhaf, Assaf A. Gilad

**Affiliations:** ^1^ Department of Chemical Engineering & Material Science Michigan State University East Lansing Michigan USA; ^2^ Michigan State University College of Human Medicine Grand Rapids Michigan USA; ^3^ Department of Computer Science and Engineering Michigan state university East Lansing Michigan USA; ^4^ Department of Chemistry Michigan State University East Lansing Michigan USA; ^5^ Department of Radiology Michigan State University East Lansing Michigan USA; ^6^ Faculty of Life Sciences Tel Aviv University Tel‐Aviv Israel; ^7^ School of Biomedical Engineering Tel Aviv University Tel Aviv Israel

**Keywords:** gadolinium‐based contrast‐agents, machine‐learning, magnetic resonance imaging, synthetic biology

## Abstract

Gadolinium‐based contrast agents (GBCAs) are indispensable tools in magnetic resonance imaging (MRI), yet their clinical use is limited by non‐specific tissue accumulation, low molecular specificity, and safety concerns. Protein and peptide scaffolds provide a promising alternative because they can bind metal ions with high selectivity and enable precise molecular targeting. However, identifying short peptide motifs with optimal gadolinium (Gd^3+^) coordination and high relaxivity remains a major challenge. Here, we used a machine‐learning‐driven peptide evolution platform, the Protein Optimization Engineering Tool (POET), to design and optimize short Gd‐binding motifs that enhance longitudinal relaxivity (*r*_1_). Two algorithmic strategies were tested: motif‐based and regular‐expression‐based representations. Both algorithms were trained on an initial set of 74 twelve‐amino‐acid peptides derived from natural EF‐hand scaffolds. Through two rounds of directed evolution and experimental screening, POET predicted peptides with up to a 24% increase in *r*_1_ ratio compared with the best natural EF‐hand. Further analysis revealed that peptides exhibiting higher relaxivity generally possessed a more negative net charge and lower isoelectric point than the buffer pH, indicating stronger electrostatic stabilization of Gd^3+^. Sequence enrichment analysis showed that acidic and small polar residues, particularly aspartic acid, glycine, and threonine, were selectively favored during evolution, while bulky hydrophobic and basic residues were depleted. These compositional trends align with improved solubility and enhanced metal coordination. Together, these results demonstrate a generalizable framework that integrates computational evolution with biophysical screening to discover new biologically derived Gd‐binding motifs. This approach provides a scalable route to engineer responsive, tunable, and biocompatible MRI contrast tags for precision imaging and molecular diagnostics.

## Graphical abstract

In this study, Dayan et al. employed a machine‐learning‐driven peptide‐evolution platform, the Protein Optimization Engineering Tool (POET), to design and optimize short Gd‐binding motifs to enhance longitudinal relaxivity (*r*_1_).

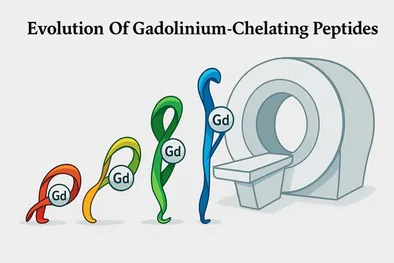

**Abbreviations**
CaBM: calcium binding motif
EF3: EF hand 3 motif
GBCA: gadolinium‐based contrast agents
pI: isoelectric point
POET: Protein Optimization Engineering Tool
PRE: paramagnetic relaxation enhancement
ProCA: Protein Contrast Agent
REE: rare earth elements
regex: regular expressions

## 1 Introduction

Gd^3+^ is a rare earth element (REE) and one of 15 lanthanide group elements. Gd^3+^'s electron configuration makes it highly reactive, and it enhances proton relaxation to create a high magnetic moment making it an ideal element for an MRI contrast agent (CA) [1, 2]. Since 1988, gadolinium‐based CAs (GBCAs) have been developed to improve stability and reduce toxicity, but they have remained the predominant choice as high‐quality contrast imaging agents [3]. The negative aspect of GBCAs is that they are a broad CA administered into the bloodstream in order to disperse throughout the entire body, which has been shown to cause other adverse effects such as accumulation in skin, muscle, bone, and brain [4, 5, 6, 7, 8]. One of the reasons Gd^3+^ accumulates in the body is that the Gd^3+^ cation, when unchelated, has biophysical characteristics similar to Ca^+2^, and several studies have investigated the potential mimicry effects this may have on body systems. Research found that Gd^3+^ ions increased mitochondrial fluidity, resulting in promotion of apoptosis in mice livers, contributing to further suspicions of unknown toxicities of Gd^3+^ in the body [9]. Previous research has also shown that the binding of GdCl_3_ to tubulin of NIH‐3T3 cells might inhibit the assembly of tubulin or depolymerize microtubules in cells, while the GBCA gadolinium diamide (gadodiamide) showed much lower levels of toxicity [10]. That being said, studies have shown GBCAs are not as inert to protein binding as many assume, demonstrating that more work must be done to understand the implications that weak but broad protein binding may have in vivo [11]. Therefore, it is extremely desirable to develop GBCAs that are more efficient, reducing the amount of potential harmful metal administered to patients.

Biologics are substances and products derived from living organisms that are used to prevent, diagnose, or treat a variety of health conditions [12]. In randomized control trials, biologics have demonstrated an ability to improve quality of life for patients suffering from joint damage, skin lesions, and gastrointestinal inflammation [13, 14]. Not only have biologics demonstrated potential to be effective pharmacological agents in chronic disease, but they also present opportunities for increased precision and tailoring to patient needs. It is highly desirable to track biologics in vivo as part of theranostics therapies, since it is important to know whether the biologic reached its target in the body. GBCAs have been used to track biologics using MRI [15], with most efforts targeting chemical conjugation of GBCA to the biologic. However, attaching Gd^3+^ chelates to antibodies, for example, often produces heterogeneous labeling, leading to variable relaxivity, impaired binding, and reduced immunoreactivity. Because each chelate contributes only modestly to the MRI signal, multiple Gd^3+^ units are required, complicating synthesis, purification, and reproducibility [16, 17].

By contrast, biologics‐based scaffolds provide a more controlled alternative. For example, lanmodulin [18], a recently discovered bacterial protein with picomolar affinity and 10^8^‐fold selectivity for lanthanides over Ca^2+^, coordinates lanthanides using EF‐hand motifs, short helix–loop–helix structures that typically bind Ca^2+^ in the calmodulin protein [19]. In lanmodulin, four EF hands have been adapted for lanthanide binding, providing a natural, high‐affinity scaffold for engineering next‐generation GBCAs. Engineered variants such as the single‐point mutated LanND‐Gd [20], achieve longitudinal relaxivity of 13.15 mM^−1^·s^−1^ at 3 T, significantly higher than that of the clinically used agent Magnevist (4.62 mM^−1^·s^−1^ at 3 T).

Another prominent biologics‐based platform is the Protein CA (ProCA) series [21], ProCA32 [22], a third‐generation protein GBCA based on calmodulin with engineered EF‐hand motifs, which achieves *r*_1_ values of ~33 mM^−1^·s^−1^ at 1.4 T and 37°C, nearly tenfold higher than Magnevist (3.3 mM^−1^·s^−1^ under identical conditions). ProCA32 can also be fused with targeting moieties (e.g., HER2, EGFR, VEGFR) without compromising metal coordination, enabling high‐sensitivity molecular imaging of cancers, fibrosis, and metastases in vivo.

Together, LanND‐Gd and ProCA32 show how protein scaffolds can deliver an order‐of‐magnitude boost in relaxivity while preserving targeting and stability. By tightly coordinating Gd^3+^ within EF‐hand motifs, these biologics minimize metal release and enable fusion with recognition domains, making them potentially powerful platforms for high‐sensitivity, clinically relevant molecular imaging.

Despite these advances, current protein‐based GBCA remain limited by the need to optimize metal‐binding motifs for maximal relaxivity in a systematic, predictive manner. Most efforts rely on single‐point mutations or rational design, which can be laborious and may miss nonintuitive sequence combinations that could further enhance performance. There is a clear need for computational tools that can efficiently explore the vast sequence space of short motifs, learn sequence–function relationships, and propose variants with improved relaxivity and binding properties.

Here we address this gap by applying POET [23] (Protein Optimization Engineering Tool), a genetic programming–based algorithm, to systematically explore and optimize lanthanide binding peptides. POET has already been successfully applied to optimize short amino acid motifs for chemical exchange saturation transfer (CEST) MRI applications [24, 25, 26], demonstrating its ability to efficiently explore sequence space and uncover nonintuitive variants with enhanced performance. POET has two versions which use different methods to recognize amino acid sequences. The original POET (called here POET_motif_ [24]) in which individuals are composed of amino acid subsequences paired with corresponding weights, and an extension of POET (called here POET_regex_ [25]) where individuals are represented by a list of regular expressions (regex) and corresponding weights.

Our objectives in the present work were to (i) discover short motifs with improved *r*_1_ relative to natural EF‐hand using a high‐throughput assay and (ii) compare POET_motif_ and POET_regex_ to assess efficiency and diversity of outputs. More broadly, we aimed to establish a pipeline for motif discovery that could inform the design of next‐generation protein‐based MRI CAs.

## 2 Methods

### 2.1 Peptide Synthesis and Preparation

The peptides were purchased from Genscript USA Inc. (Piscataway, NJ). HEPES (4‐(2‐hydroxyethyl)‐1‐piperazineethanesulfonic acid) and GdCl_3_ were purchased from Thermo Scientific Chemicals, Waltham, MA USA. HEPES was chosen because other buffers containing phosphates tend to form Gd‐phosphate sediments.

### 2.2 MRI Measurements

The ability of a CA to produce MRI contrast is quantified by relaxivity, which is the CA's ability to change the relaxation rate of a solution as a function of its concentration. To measure relaxivity one must measure the longitudinal relaxation time (T_1_) of several dilutions of the CA and extract the slope from linear regression of the following equation

(1) $$1/T_{1}=1/T_{1}^{0}+r_{1}\times \left[\mathrm{CA}\right]$$

where $T_{1}$ and $T_{1}^{0}$ are the longitudinal relaxation time of the solution with and without the CA respectively. *r*_1_ is the longitudinal relaxivity and [CA] is the GdCl_3_ concentration of the CA measured by inductively coupled plasma (ICP) (Varian 710‐ES). ICP samples were prepared by nitric acid digestion.

Peptides were dissolved in HEPES 50 mM, and GdCl_3_ was added to a final concentration of 0.2 mM. Addition of GdCl₃ lowered the solution pH from approximately 7.5 to 5.3; therefore, peptide–Gd samples were incubated and measured at pH 5.3. Samples were incubated for 24 h before MRI measurements.

Because relaxivity depends on experimental conditions, including magnetic field strength, temperature, buffer composition, and Gd^3+^ speciation, the *r*_1_‐ratio assay was used only for relative comparison among peptide–Gd samples measured under matched conditions. Practical considerations, including time and material requirements, further supported the use of this relative screening strategy.

For absolute relaxivity measurements, selected peptide–Gd samples were dialyzed after the 24‐h incubation step to reduce unbound Gd^3+^. Samples (0.5 mL) were transferred into Biotech ce dialysis tubing with a 100–500 Da molecular weight cutoff and 10 mm flat width (Repligen, SKU 131048), closed with dialysis clips, and submerged in approximately 200 mL of 50 mM HEPES buffer on a table shaker, corresponding to an approximate sample:buffer volume ratio of 1:400. After 24 h, the dialysis buffer was replaced with fresh 50 mM HEPES, and samples were dialyzed for an additional 42 h. Post‐dialysis Gd^3+^ concentrations were quantified by ICP and are reported in Table 1. Because free Gd^3+^ is expected to diffuse through the dialysis membrane during the 66‐h dialysis protocol and buffer exchange, the retained Gd^3+^ is expected to be enriched in peptide‐associated metal. However, dialysis does not establish complete removal of unbound Gd^3+^, and residual free Gd^3+^ may remain and contribute to the measured absolute *r*_1_ values.

**TABLE 1:** Comparison of absolute *r*_1_ to *r*_1_ ratio.

Source	Peptide sequence	*r*_1_ ratio (A.U.)	Absolute *r*_1_ (mM^−1^ s^−1^)	SE/SD	*n*	Post dialysis Gd^3+^ concentration (mM)
CaBM [27]	DKDGNGYISAAE	0.91	14.97	1.30	1	19.21 ± 0.93
POET regex1	NSAMADQADDDP	1.03	8.63	5.40	2	12.58 ± 7.93
Lanthanide binding [28]	DKDGDTIDEREI	1.35	23.10	0.80	1	18.28 ± 4.89
EF 1 hand motif [29]	DPDKDGTIDLKE	1.60	22.26	0.63	1	23.13 ± 1.05
POET Motif 1	DMMHDKTDCNDT	1.71	24.33	0.85	1	19.33 ± 0.39
EF3 [29]	DPDNDGTLDKKE	1.85	19.80	0.42	1	31.75 ± 1.88
POET regex 2	SDDNHSDLGDDL	2.14	33.80	0.38	1	58.86 ± 2.91
POET regex 2	TQDSDDGMEDED	2.16	35.48	0.19	1	47.87 ± 2.32
POET motif 2	GDDEDDQCHQDG	2.17	26.26	0.22	1	71.37 ± 2.66
POET regex 1	IDCRGTEDDDPN	2.30	31.94	0.39	1	53.15 ± 1.54

To create a series of dilutions, an epMotion 5073 liquid handler was used to ensure pipetting accuracy. The sample, along with six or seven sample dilutions and a blank solution, were pipetted into a 396 plate cut to fit inside the MRI magnet bore, sample volume inside the wells was ~100 μL. Seven columns along the phase axis and 5 rows along the read axis could be measured with a relatively homogeneous field. Two custom fitted plates were stacked on top of each other, enabling measurements of two slices simultaneously.

MRI measurements were performed on a horizontal 30‐cm bore 7 T Bruker preclinical MRI with ParaVision 360 v3.2 on a Linux computer running CentOS7, using a B‐GA12SHP high performance gradient insert with built‐in shims. A variable repetition time T_1_ map was acquired using a RARE sequence with 9 TRs (33, 122, 297, 497, 727, 1577, 2777, 4809, and 12496) ms/TE 6.88 ms, RARE factor = 1, FOV ~ 40 × 25 mm, with the matrix adjusted to ensure a spatial resolution of 0.25 × 0.25 mm, slice thickness = 1.2 mm, one or two slices; echo spacing was 6.88 ms. This is correlated to measurement times of 40 to 60 min. Finally, the T_1_ for each well was measured using the image sequence analysis module in Paravision, then *r*_1_ ratio or absolute *r*_1_ relaxivity were calculated [30].

### 2.3 Peptide Evolution Using Machine Learning

Two versions of the POET algorithm [24] were compared for their ability to create peptides that can shorten the T_1_ relaxation of water and have higher *r*_1_ ratio. POET_motif_ and POET_regex_, the former using motifs represented as substrings, and the latter using regex, a formal way of defining a set of character sequences [31]. A motif/regex was classified as beneficial when it had *r*_1_ ratio > 1, which corresponds to the *r*_1_ ratio of GdCl_3_ without a peptide. POET uses a form of supervised training called Genetic Programming. Each POET trial generates a set of rules that determine *r*_1_ ratio by checking the composition of a proposed peptide sequence. If a given motif (or regex) is found, the corresponding weight is added to a sum value, in this case *r*_1_ ratio. The rulesets interact through recombination and mutation over timesteps (called *generations*), and once all generations are completed, the most accurate model is returned for peptide generation. Both POET variants were trained with 74 12‐amino acid long peptides incubated with GdCl_3_. The *r*_1_ of the peptides was normalized to the *r*_1_ of the buffer with the same amount of GdCl_3_.

### 2.4 Nuclear Magnetic Resonance (NMR) Measurements

Solution NMR experiments were carried out on a 600‐MHz Bruker Avance NEO NMR spectrometer equipped with a 5‐mm Prodigy TCI cryoprobe. ^1^H NMR experiments were run at 25°C using the vendor supplied water suppression pulse sequence (noesygppr1d) to measure ^1^H spectra of 1 mM peptide of EF3 and CaBM [27]. Both peptides were suspended in HEPES 50 mM with 10% D_2_O for locking, shimming, and chemical shift referencing with or without 5‐μM GdCl_3_.

## 3 Results and Discussion

### 3.1 Peptide Evolution

**FIGURE 1:**
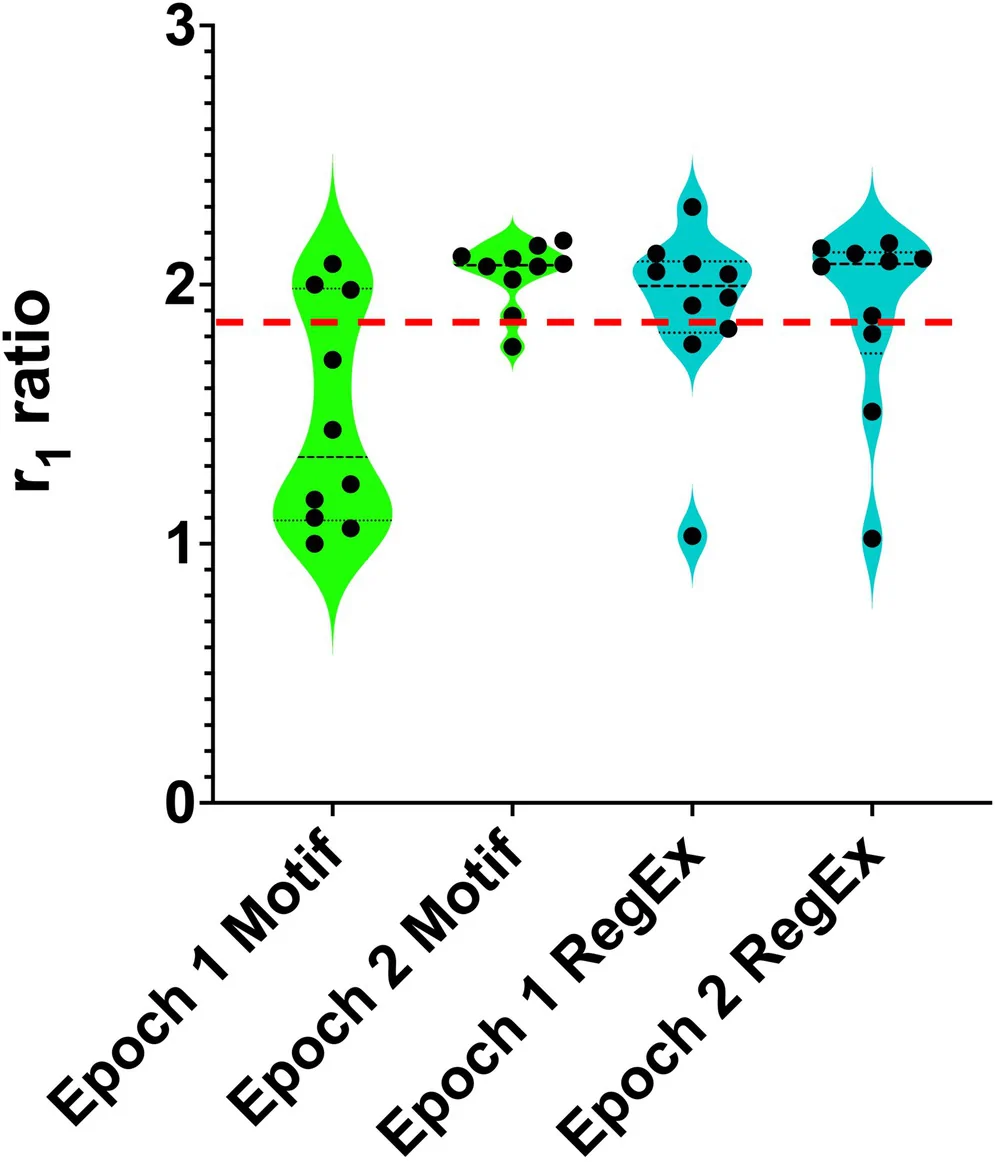
*r*_1_ ratio of peptides predicted by POET_motif_ and POET_regex_ across Epochs 1 and 2. Values correspond to the *r*_1_ ratios reported in Table S1: The mean *r*_1_ ratio is shown when independent repeat measurements were available, and the single measured *r*_1_ ratio is shown when no repeat measurements were available. Error estimates and replicate information are reported in Table S1. The red dashed line represents the *r*_1_ ratio of the EF 3 hand motif (EF3; *r*_1_ ratio = 1.85), the best‐performing peptide in the initial dataset.

Both POET algorithms were applied to evolve Gd‐binding peptides with enhanced MRI relaxivity. Figures 1 and S3 show plots of the *r*_1_ ratio of the peptides predicted by POET_motif_ and POET_regex_ over two POET cycles dubbed Epoch 1 and 2. In Figure 1 the red dashed line represents the *r*_1_ ratio of EF hand 3 motif (EF3) (*r*_1_ = 1.85), a 12‐amino‐acid from Lanmodulin that showed the highest *r*_1_ ratio from the training data. For the POET_motif_ in Epoch 1, only three peptides showed better contrast than the EF3 peptide; however, in Epoch 2, 9 of 10 peptides surpassed the *r*_1_ ratio of the EF3 hand peptide. For POET_regex_ 7 out of 10 peptides surpassed the *r*_1_ ratio of EF3 in both Epochs 1 and 2. The best motif found had an *r*_1_ ratio of 2.3, a 24.3% improvement over the EF3. Peptides displaying an *r*_1_ relaxivity ratio lower than that of EF3 suggest that POET did not have sufficient data to fully explore the sequence space. Nevertheless, the algorithm successfully predicted novel peptides with enhanced relaxivity, demonstrating its capacity to discover functionally improved motifs beyond those found in nature. Further model refinement with additional experimentally measured epochs may improve the relationship between POET prediction scores and measured *r*_1_ ratio values.

Small dataset size is a general limitation for biological and chemical machine learning applications that rely heavily on statistical inference, such as neural networks, which require estimating many continuous parameters. In contrast, the Genetic Programming approach used here operates on discrete structures and can generate compact models that capture underlying relationships without extensive data, for example, by deriving explicit mathematical expressions rather than high‐dimensional approximations. This reduces overfitting risk and makes it particularly well‐suited to small datasets, such as those typical in peptide design [32]. Given the time‐ and cost‐intensive nature of peptide synthesis and characterization, the available dataset was used to iteratively improve peptide function while refining the predictive model.

### 3.2 Isothermal Titration Calorimetry

To directly evaluate Gd^3+^ binding under conditions relevant to this study, we performed isothermal titration calorimetry (ITC) with the lanmodulin‐derived EF3 peptide, DPDNDGTLDKKE. Isolated lanmodulin EF‐hand peptides were previously shown to bind representative lanthanides in the micromolar range while showing no detectable Ca^2+^ binding [29]; however, those measurements did not include Gd^3+^. Consistent with these reports, titration of GdCl₃ into EF3 produced a clear binding isotherm, yielding an apparent Kd of 1.54 μM (Figure S1A), whereas CaCl_2_ titration under comparable conditions did not produce a measurable binding isotherm (Figure S1B). The lower affinity of the isolated peptide compared with full‐length *Methylobacterium extorquens* lanmodulin is expected: isolated EF‐hand loops bind lanthanides in the micromolar range, whereas full‐length lanmodulin binds Gd^3+^ with an apparent Kd of approximately 10^−11^ M, likely because the full protein provides a folded EF‐hand environment and multiple metal‐binding sites that are absent from the flexible 12‐residue peptide [18].

### 3.3 Validation of the Gd Relaxivity

The *r*_1_ ratio is a convenient high‐throughput metric reflecting the ability of Gd‐binding peptides to enhance longitudinal relaxivity; however, it represents combined contributions from peptide‐bound and free Gd^3+^ ions. To qualitatively assess whether the Gd^3+^ spectral environment differed before and after dialysis, we performed electron spin resonance (ESR) spectroscopy (Figure S2). The dialyzed EF3 sample exhibited a broad, downfield‐shifted, low‐intensity ESR signal consistent with a Gd^3+^ environment that differs from freely dissolved Gd^3+^. In contrast, the undialyzed EF3 + Gd^3+^ mixture, after subtraction of the free Gd^3+^ spectrum, displayed a strong residual central peak and a downfield shoulder. The central peak reflects an incomplete cancelation of the intense free Gd transition, while the shoulder is consistent with a heterogeneous population of Gd^3+^ interacting with the peptide. The distinct downfield positions of the two traces suggest that the Gd^3+^ species present before and after dialysis occupy slightly different electronic environments due to differences in exchange, hydration, and coordination equilibrium. These ESR results suggest that undialyzed samples contain a fraction of free Gd^3+^ with a different electronic environment than the peptide‐associated species observed after dialysis. Because this ESR analysis was qualitative and was not used to quantify bound and free Gd^3+^ populations, these spectra were interpreted as supportive evidence rather than as a quantitative measurement of the different Gd^3+^ species present. Therefore, we measured absolute *r*_1_ after dialysis to evaluate whether it correlates with the *r*_1_ ratio used in the initial screen.

**FIGURE 2:**
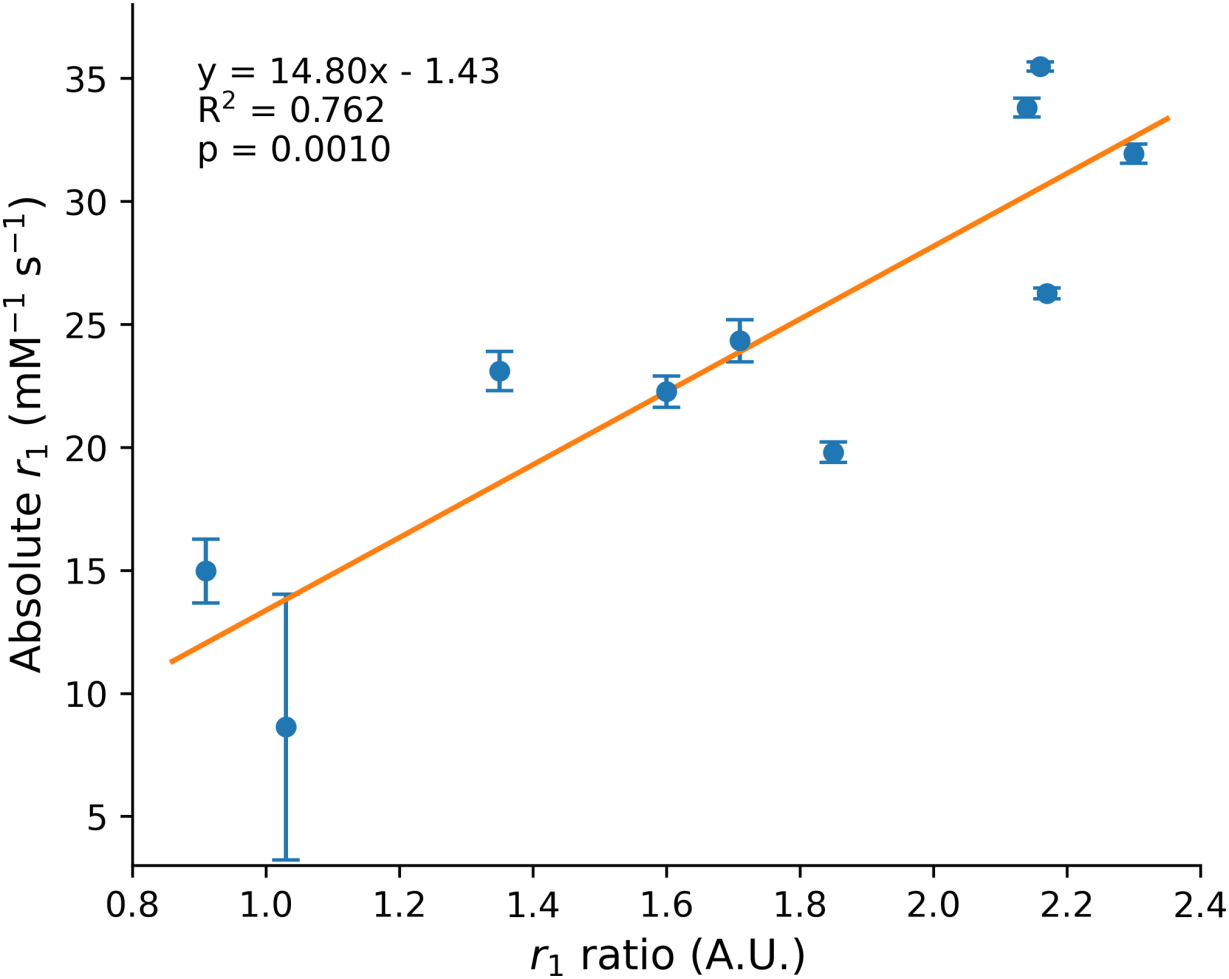
Correlation between *r*_1_ ratio (A.U.) and absolute longitudinal relaxivity (*r*_1_, mM^−1^ s^−1^) measured after dialysis to reduce unbound Gd^3+^. Data correspond to the peptide values reported in Table 1. Error bars represent uncertainty in the absolute *r*_1_ measurements: SE is the standard error of the fitted slope from the dilution‐series regression for single measurements (*n* = 1), whereas SD is the standard deviation across independent measurements when *n* = 2. Ordinary least‐squares linear regression was used to evaluate the relationship between *r*_1_ ratio and absolute *r*_1_. Linear regression shows that the high‐throughput screening metric correlates with absolute relaxivity (*R* ^2^ = 0.762, *p* = 0.0010).

With this rationale, the absolute relaxivities (*r*_1_) of Gd‐binding peptides were measured after two cycles of dialysis totaling 66 h against HEPES buffer to reduce unbound Gd^3+^. Dialysis typically reduced the total Gd^3+^ concentration from 0.2 mM to ~35 μM (Table 1); preliminary measurements performed using a shorter 48‐h dialysis protocol were not included because they showed higher retained Gd^3+^ concentrations. Peptides showing a range of *r*_1_ ratios were selected, including EF‐hand motifs, a calcium‐binding motif (CaBM) peptide, a lanthanide‐binding peptide, and peptides predicted by POET (Table 1). Post‐dialysis Gd^3+^ concentrations were quantified by ICP and are reported in Table 1. For samples with repeated ICP measurements from the same sample, Gd^3+^ concentrations were averaged before calculating a single absolute *r*_1_ value. A positive correlation was found between the *r*_1_ ratio and absolute *r*_1_ (Figure 2; *R* ^2^ = 0.762, *p* = 0.0010). These findings support the use of the *r*_1_ ratio as a practical high‐throughput screening metric for identifying peptide–Gd samples with increased absolute relaxivity. Taken together, these results demonstrate that POET can predict peptide sequences with improved Gd‐associated MRI contrast under the screening conditions used here.

Values represent samples prepared using the dialysis protocol. The “*n*” column refers to the number of independent MRI/dialysis measurements used for absolute *r*_1_ analysis. For *n* = 1, SE/SD reports the standard error of the fitted slope from the dilution‐series regression. For *n* = 2, SE/SD reports the standard deviation across two independent measurements. Repeated ICP measurements of the same MRI/dialysis sample were averaged before calculating absolute *r*_1_ and were not counted as independent replicates.

### 3.4 Analysis of Peptides' Isoelectric Point and Net Charge

**FIGURE 3:**
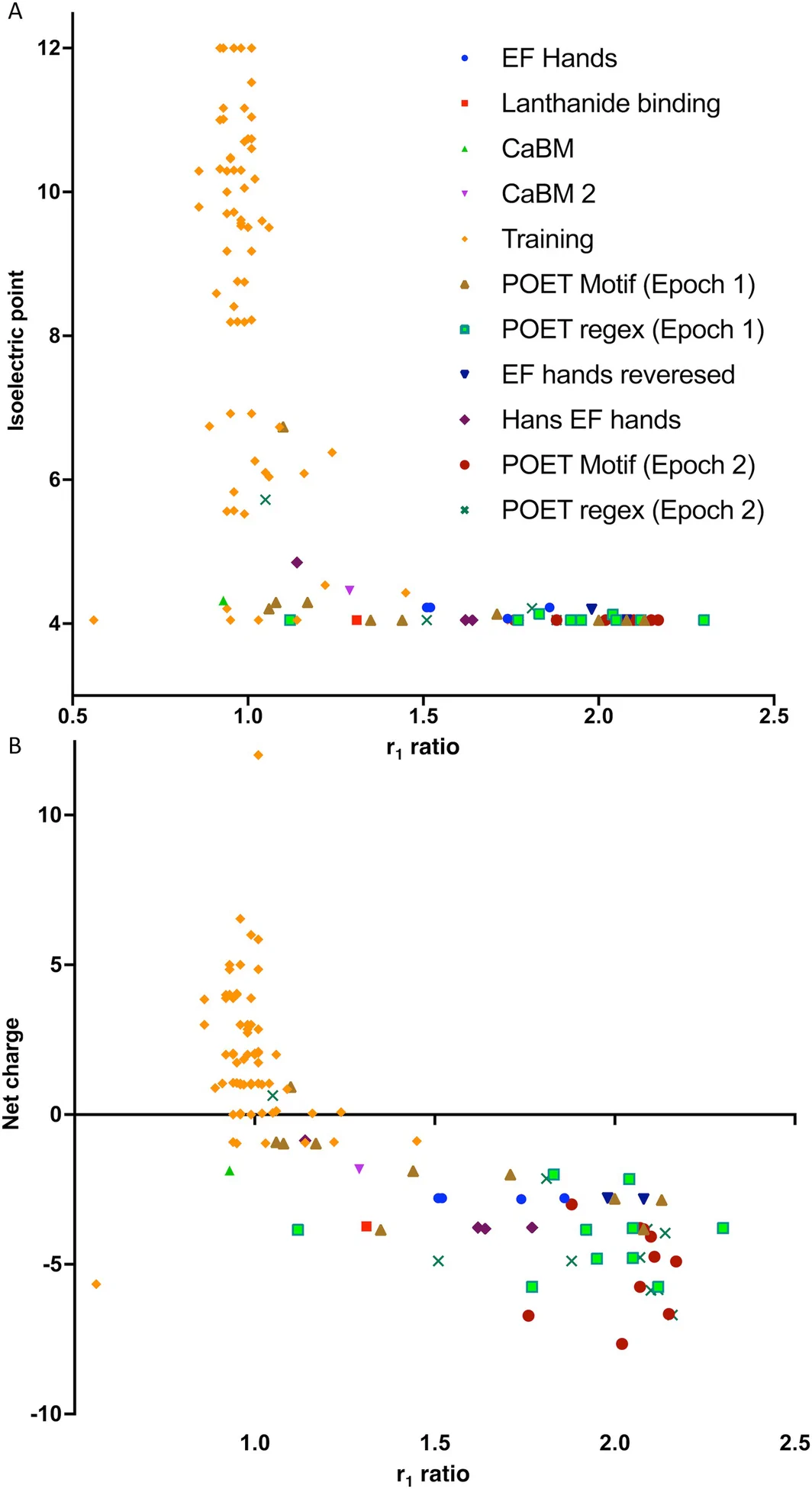
(A) Calculated net charge of a peptide plotted against its *r*_1_ ratio. (B) Calculated isoelectric point (pI) of a peptide plotted against its *r*_1_ ratio. Net charge and pI were interpreted in the context of the experimental screening pH of 5.3. The different groups are listed in Table S1.

After two Epochs, an extensive analysis was performed to evaluate POET's predicted peptides. First, the net charge and the isoelectric point (pI) at pH 5.3 were calculated for each peptide and plotted against their *r*_1_ ratio. The results in Figure 3 show that most peptides with an *r*_1_ ratio better than free Gd had a negative net charge, and usually, the *r*_1_ ratio improved with more negative net charge. This trend is consistent with favorable electrostatic interaction between negatively charged peptide side chains and Gd^3+^. For the pI analysis, peptides with pI values below the experimental pH of 5.3 generally showed higher *r*_1_ ratios, consistent with these peptides carrying net negative charge under the screening conditions.

Importantly, because the assay was performed at pH 5.3, this analysis may preferentially favor acidic peptides that remain negatively charged under these conditions. The ranking of peptide performance may differ at physiological pH, where peptide charge state, Gd^3+^ speciation, and buffer interactions may change. Therefore, the observed relationship between low pI, negative net charge, and higher *r*_1_ ratio should be interpreted within the context of the pH 5.3 screening assay. Future optimization of these motifs for biological applications should include screening and validation under physiological buffer and pH conditions.

The results suggest that for further evolution, the peptides' net charge should be negative, and their pI should be lower than the buffer's pH, both conditions easily screened using POET. It is possible that some motifs and regex were neglected by POET because the net charge of the molecule was too high, so it could be beneficial for future Epochs to use only negatively charged peptides. However, buffer pH should be chosen based on the intended application and Gd^3+^ compatibility rather than solely to improve screening performance.

### 3.5 Paramagnetic Relaxation Enhancement (PRE)

PRE measurements were performed to validate that Gd^3+^ binds to the peptides and to investigate which amino acids are more involved in the binding. A comparison was made between similar EF‐hands, EF3 [29], known to bind lanthanides and CaBM [27], known to bind Calcium.

**FIGURE 4:**
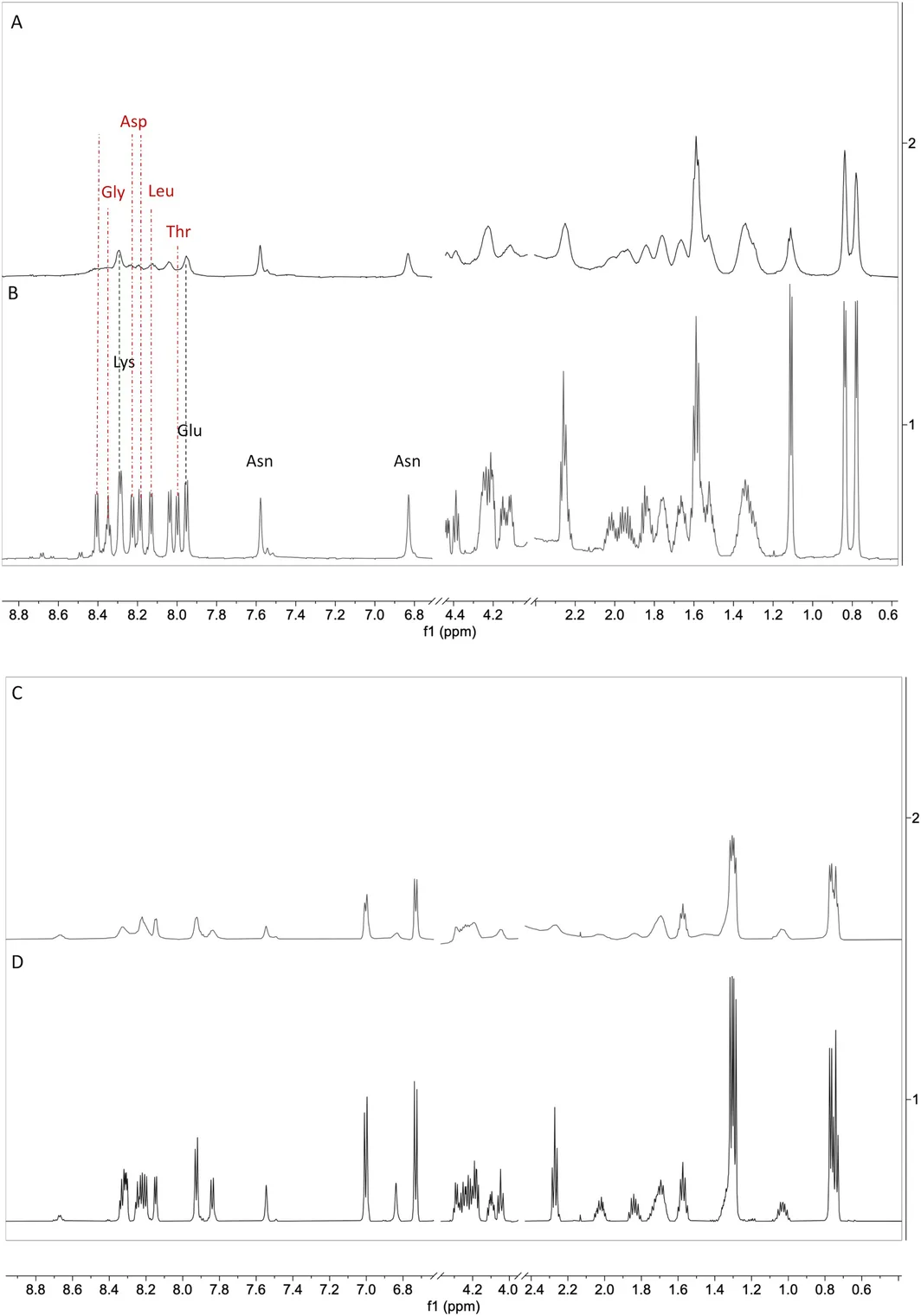
^1^H NMR spectra of (A) EF3 DPDNDGTLDKKE (1‐mM peptide) with 5‐μM GdCl_3_. (B) EF3 (1‐mM peptide). The vertical lines connect between the peaks to show the line broadening when there's presence of GdCl_3_. The red dot‐dashed line connects the peaks that were broadened to a higher extent, while the black dashed line connects the peaks that were less broadened. (C) The Ca binding motif (CaBM) DKDGNGYISAAE (1 mM peptide) with 5‐μM GdCl_3_ and (D) CaBM (1‐mM peptide).

Since Gd^3+^ is a paramagnetic ion that significantly shortens both the T_1_ and T_2_ [33] of any NMR active nuclei within approximately 25 Å of the binding site, NMR was used to measure ^1^H spectra of 1 mM peptide of EF3 and CaBM with or without 5 μM GdCl_3_. As shown in Figure 4, EF3 displays a more significant increase in the linewidths of all proton peaks with a concomitant reduction in intensity due to paramagnetic relaxation than those of CaBM. PRE will be more pronounced for those protons proximal to the binding site than for those distal, which is why EF3 displays more significant broadening of the NH peaks of Asp‐D, Gly‐G, Leu‐L, and Thr‐T (7.9–8.4 ppm) than other proton resonances in the peptide. CaBM also displays broadening for Asp‐D, Gly‐G, Ser‐S, and Glu‐E, but to a lesser extent, and there is comparatively little relaxation enhancement for the aliphatic protons of leucine (0.95 and 1.6 ppm) thereby indicating that these protons are more distal to the Gd^3+^ binding sites. The number of NH resonances broadened into the baseline is higher for EF3 than CaBM which can be attributed to a higher percentage of amino acids binding to Gd^3+^ for EF3 than CaBM.

### 3.6 Amino Acid Distribution

**FIGURE 5:**
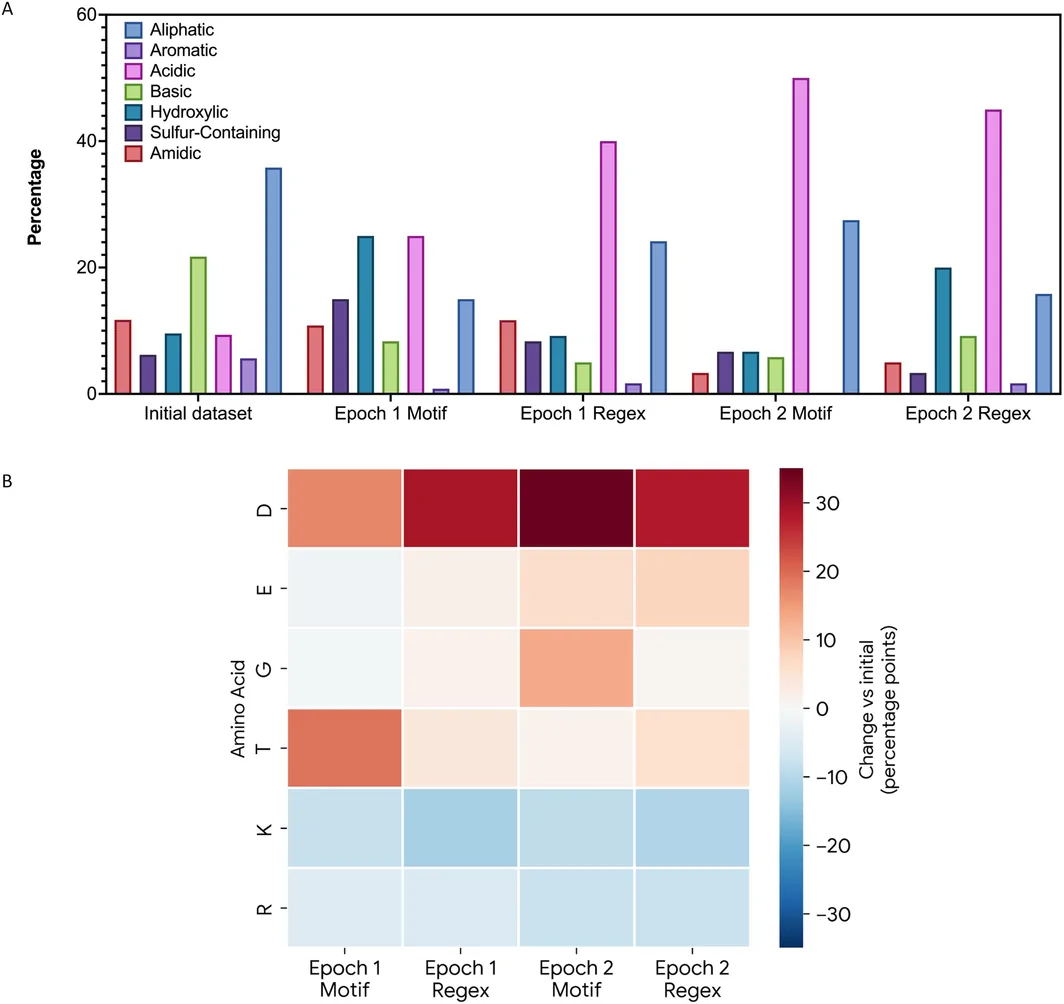
The amino acid distribution in the training data, Epoch 1 and 2, with either motif or regular expressions (regex) POET. (A) Column bar representation of the amino acid categories distribution across the datasets. (B) Heatmap showing the change in selected amino acid frequencies relative to the initial training dataset, reported in percentage points. The selected residues highlight the main compositional trends observed during POET evolution. The full 20‐amino‐acid percentage heatmap with numerical values is provided in Figure S4.

The performances of POET_motif_ and POET_regex_ were evaluated and compared by the amino acid composition of each algorithm and Epoch. Figure 5A shows the distribution of the different amino acid classes within the resulting peptide sequences, Acidic (D, E), Basic (K, H, R), Aliphatic (A, G, I, L, P, V), Aromatic (F, W, Y), Hydroxylic (S, T), Sulfur‐containing (C, M) and Amidic (N, Q). Figure 5B highlights selected amino acids most relevant to the compositional trends observed during POET evolution, plotted as the change in percentage points relative to the initial training dataset. The full 20‐amino‐acid percentage heatmap, including numerical values, is provided in Figure S4. Importantly, the initial training dataset exhibited a nearly uniform distribution of amino acid residues, providing an unbiased starting point for the evolution performed by POET.

Across all groups, there was a consistent and pronounced enrichment of acidic residues (particularly aspartic acid, D) and a marked depletion of basic amino acids (notably lysine, K, and arginine, R). This suggests a strong selection for negatively charged residues capable of coordinating with the positively charged Gd^3+^ ions and simultaneous avoidance of positively charged residues to minimize electrostatic repulsion. Aspartic acid emerged as the dominant residue in both motif and regex patterns during both evolutionary epochs, comprising over 24% of amino acids in all of POET's outputs. This selection mirrors the composition of many natural EF‐hand motifs, which often contain multiple aspartic acids involved in metal ion coordination.

While there was a general reduction in non‐polar aliphatic residues, one exception was glycine (G), which increased significantly in Epoch 2 motifs. Glycine's small side chain likely contributes to increased peptide flexibility and solubility, properties that may be advantageous in a hydrophilic or dynamic binding context. The hydroxylic class (serine and threonine) showed moderate enrichment, particularly threonine (T), which rose sharply in Epoch 1 Motif. These residues support hydrogen bonding and surface polarity, reinforcing the hydrophilic nature of the evolving peptides.

The aromatic and basic classes, especially phenylalanine (F), tryptophan (W), and lysine (K), were consistently depleted across all outputs, indicating a preference against bulky, hydrophobic, or positively charged side chains. Sulfur‐containing and amidic residues (C, M, N, Q) exhibited more subtle variations, with no strong trend in either direction.

Consistent with these compositional trends, PRE measurements confirmed that several of the amino acids enriched by POET are indeed directly involved in Gd^3+^ coordination. In particular, residues such as aspartic acid (D), glycine (G), and threonine (T), which showed strong enrichment in the evolved sequences, were among those that exhibited significant signal broadening in EF‐hand peptides upon Gd^3+^ binding.

Notably, the inclusion of all 20 amino acids in the training set allowed POET to explore a broad sequence space during motif and regex discovery, enabling the algorithm to converge on a highly specific and chemically coherent set of residue preferences aligned with metal‐binding functionality.

## 4 Conclusions

We applied POET to expand and optimize short lanthanide‐binding peptide motifs and demonstrate a practical, data‐driven route to improve Gd‐mediated T_1_ contrast. Starting from an experimental library of 74 sequences and using iterative POET cycles, POET predicted novel peptides with improved *r*_1_ ratios, with the best POET‐predicted peptide reaching an *r*_1_ ratio of 2.30 (~24% higher than the best initial EF‐hand, EF3). The screening metric (*r*_1_ ratio) correlated with absolute relaxivity (*R* ^2^ ~ 0.762, *p* ~ 0.0010), supporting its use as a high‐throughput screening approach, and PRE spectra support that Gd^3+^ is in proximity to peptide residues. Sequence analysis revealed strong enrichment of acidic residues (notably Asp), consistent with electrostatic and coordinating roles in Gd^3+^ binding.

These findings show that coupling POET with a high‐throughput *r*_1_ screening can efficiently explore short‐motif sequence space and produce superior candidates; and that running complementary POET recognition modes (motif and regex) yields both rapid consensus improvements and broader, nonintuitive solutions. Some discovered peptides could be evaluated further as standalone contrast‐agents candidates. Future work should validate motif performance under physiological buffer and pH conditions, perform additional replicated absolute *r*_1_ measurements and evaluate motif transferability into full proteins (e.g., Lanmodulin) and biologics (e.g., antibodies). Overall, POET combined with a high‐throughput pipeline provides the foundation for designing peptide tags and for guiding the development of next‐generation biologics‐based Gd^3+^ MRI CAs with improved sensitivity and translational potential.

## Author Contributions

Assaf A. Gilad, Wolfgang Banzhaf, and Nir A. Dayan conceived the study. Assaf A. Gilad, Wolfgang Banzhaf, and Nir A. Dayan designed the experiments. Nir A. Dayan, Nicolas Scalzitti, Iliya Miralavy, Daniel Holmes, and Mark Kocherovsky performed experiments and collected data. Nir A. Dayan, Makayla Long, Nicolas Scalzitti, Iliya Miralavy, Daniel Holmes, Mark Kocherovsky Wolfgang Banzhaf, and Assaf A. Gilad analyzed and interpreted the data. Assaf A. Gilad and Wolfgang Banzhaf supervised the project. Nir A. Dayan, Makayla Long, Assaf A. Gilad, and Wolfgang Banzhaf wrote the original draft of the manuscript, and all authors reviewed and edited the manuscript. All authors approved the final version of the manuscript.

## Funding

This work was supported by the National Institutes of Health/National Institute of Biomedical Imaging and Bioengineering (R01‐EB031008 and R01‐EB031936 to A.A.G.; R01‐EB030565 to A.A.G. and W.B.).

## Conflicts of Interest

The authors declare no conflicts of interest.

## Supporting information


**Figure S1:** Isothermal titration calorimetry analysis of EF3 peptide binding to Gd^3+^ and Ca^2+^. **(A)** GdCl₃ (600 μM) was titrated into EF3 peptide (DPDNDGTLDKKE; 20 μM) in 50 mM HEPES using a MicroCal VP‐ITC. The upper panel shows the thermogram and the lower panel shows the integrated heats fitted using a single‐site hetero‐association model, yielding an apparent Kd = 1.54 μM. **(B)** CaCl_2_ titration into EF3 under comparable conditions showed no clear binding isotherm and was therefore not fitted. The Ca^2+^ control supports preferential interaction of the isolated EF3 motif with trivalent Gd^3+^ over Ca^2+^ under these assay conditions.
**Figure S2:** Electron spin resonance (ESR) spectra of EF 3 hand motif (EF3) peptide incubated with Gd^3+^ under two conditions. The black trace represents EF3 (740 μM) incubated with 1 mM GdCl₃ followed by dialysis to reduce unbound metal. The blue trace shows the spectrum subtraction of EF3 (740 μM) incubated with 250 μM GdCl₃ without dialysis minus free Gd^3+^ at the same concentration. Measurements were performed in 50 mM HEPES at 130 K using X‐band continuous‐wave ESR. The dialyzed sample exhibits lower intensity and broader features, consistent with a change in the Gd^3+^ spectral environment after dialysis.
**Table S1:** Complete list of peptides.
**Figure S3:** *r*_1_‐ratio values for the initial dataset and POET‐predicted peptides. GdCl₃ without peptide was used as the reference control, and higher *r*_1_‐ratio values indicate greater apparent MRI contrast under the screening conditions. Error bars represent the propagated standard error (SE) of the *r*_1_ ratio calculated from the fitted slopes of the peptide–Gd and peptide‐free GdCl₃ dilution curves. Orange bars indicate peptides with *r*_1_‐ratio values equal to or higher than EF3, the best‐performing peptide in the initial dataset. The complete dataset is provided in Table S1.
**Figure S4:** Amino acid percentage heatmap corresponding to Figure 5B. Numerical values are shown in each cell to provide the full amino acid composition of the initial training dataset and the POET‐predicted peptide groups.

## Data Availability

POET's code can be accessed freely at https://gitlab.com/NicolasScalzitti/poet_regex and https://github.com/elemenohpi/POET.
