# Supplementary material for: Machine Learning‐Based Development of Gadolinium Binding Peptides
Source: NMR Biomed. 2026 Sep 11;39(10):e70381. doi: 10.1002/nbm.70381 (PMC13569970; doi:10.1002/nbm.70381)
Supplement: Supplementary file 1 [file NBM-39-e70381-s001.docx]

### Machine learning-based development of Gadolinium binding peptides

Nir A. Dayan^1^, Makayla Long^2^, Nicolas Scalzitti^1,3^, Iliya Miralavy^3^, Daniel Holmes^4^, Mark Kocherovsky^3^, Wolfgang Banzhaf^3^*, and Assaf A. Gilad^1,5-7^*

^1^ Department of Chemical Engineering & Material Science, Michigan State University, East Lansing, Michigan 48824, USA

^2^ Michigan State University College of Human Medicine, Grand Rapids, Michigan 49503, USA

^3^Department of Computer Science and Engineering, Michigan state university, 48824 East Lansing, Michigan 48824, USA

^4^Department of Chemistry, Michigan State University, East Lansing, Michigan 48824, USA

^5^Department of Radiology, Michigan State University, East Lansing, Michigan 48824, USA

^6^Faculty of Life Sciences, Tel Aviv University, Tel-Aviv, Israel

^7^The Scojen Institute for Synthetic Biology, Reichman University, Herzliya, Israel

*Corresponding authors:

[assaf.a.gilad@gmail.com](mailto:assaf.a.gilad@gmail.com)

[banzhafw@msu.edu](mailto:banzhafw@msu.edu)

Isothermal titration calorimetry of EF3–Gd³⁺ binding

Isothermal titration calorimetry was performed using a MicroCal VP-ITC. The sample cell was washed with 30 mL of 50 mM HEPES buffer before loading. EF3 peptide, DPDNDGTLDKKE, was prepared at 20 µM in 50 mM HEPES, and 1.5 mL was loaded into the cell. GdCl₃ was prepared at 600 µM in the same buffer and loaded into the syringe. GdCl₃ was titrated into EF3, and heats of injection were recorded (figure S1A). As a control, CaCl₂ was titrated into EF3 under comparable conditions (figure S1B).	Raw thermograms were integrated and analyzed using the NITPIC/SEDPHAT/GUSSI workflow^1^. The Gd³⁺ titration was fitted using a single-site hetero-association model, yielding an apparent dissociation constant Kd of 1.54 µM. The Ca²⁺ titration did not produce a clear binding isotherm and was therefore not fitted. Because the Gd³⁺ ITC measurement was performed as a single titration, no replicate-derived uncertainty is reported.

**Figure S1.** Isothermal titration calorimetry analysis of EF3 peptide binding to Gd³⁺ and Ca²⁺. **(A)** GdCl₃ (600 µM) was titrated into EF3 peptide (DPDNDGTLDKKE; 20 µM) in 50 mM HEPES using a MicroCal VP-ITC. The upper panel shows the thermogram and the lower panel shows the integrated heats fitted using a single-site hetero-association model, yielding an apparent Kd=1.54 µM. **(B)** CaCl₂ titration into EF3 under comparable conditions showed no clear binding isotherm and was therefore not fitted. The Ca²⁺ control supports preferential interaction of the isolated EF3 motif with trivalent Gd³⁺ over Ca²⁺ under these assay conditions.

Electron spin resonance measurements

Electron spin resonance (ESR) spectroscopy^2^ was used to qualitatively compare the spectral features of Gd³⁺ in EF3 samples before and after dialysis. EF3 peptide (740 µM) was incubated with GdCl₃ under two conditions: (i) 1 mM GdCl₃ followed by dialysis to reduce unbound metal and (ii) 250 µM GdCl₃ without dialysis. Measurements were performed in 50 mM HEPES at 130 K on an X-band continuous-wave ESR spectrometer. Figure S2 shows the comparison of these spectra. The black trace represents EF3 incubated with Gd³⁺ and subsequently dialyzed to reduce free ions, while the blue trace corresponds to the spectrum subtraction of (undialyzed EF3 incubated with Gd³⁺) minus (free Gd³⁺ spectrum of the same concentration). Thus, the figure does not display the raw undialyzed EF3 or free Gd³⁺ spectra individually, but rather the subtraction spectrum used to highlight spectral features not fully accounted for by free Gd³⁺ alone. The dialyzed sample (black trace) exhibits a broad, downfield shifted low-intensity feature consistent with a Gd³⁺ environment that differs from freely dissolved Gd³⁺. A small central peak remains, which may reflect residual free or weakly associated Gd³⁺ after dialysis.

The subtracted spectrum (blue trace) retains both a strong central peak and a downfield shoulder. The intense central peak results from incomplete cancellation of the free-Gd transition, which can differ in amplitude or linewidth between samples and therefore does not fully subtract. The downfield shoulder is consistent with Gd³⁺ interacting with the peptide in the undialyzed mixture. This signal may differ from the dialyzed species; it is more heterogeneous, influenced by higher free-Gd concentration and dynamic exchange with bulk solvent, leading to a slightly different ESR position and shape. Together, these data support a change in the observed Gd³⁺ spectral environment after dialysis and are consistent with enrichment of peptide-associated Gd³⁺ species in the dialyzed sample. Because this ESR analysis was qualitative and was not calibrated to quantify the amount of bound versus free Gd³⁺, these spectra were interpreted as supportive evidence for changes in the Gd³⁺ environment rather than as a quantitative measurement of peptide–Gd³⁺ binding.

**Figure S2.** Electron spin resonance (ESR) spectra of EF 3 hand motif (EF3) peptide incubated with Gd³⁺ under two conditions. The black trace represents EF3 (740 µM) incubated with 1 mM GdCl₃ followed by dialysis to reduce unbound metal. The blue trace shows the spectrum subtraction of EF3 (740 µM) incubated with 250 µM GdCl₃ without dialysis minus free Gd³⁺ at the same concentration. Measurements were performed in 50 mM HEPES at 130 K using X-band continuous-wave ESR. The dialyzed sample exhibits lower intensity and broader features, consistent with a change in the Gd³⁺ spectral environment after dialysis.

Peptide dataset and results

Table S1 contains the entire dataset used in this study. Peptides 1 to 74 were used as the initial training for POET predictions, dubbed Epoch 1. POETmotif Epoch 1 predictions correspond to peptides 75-84, and POETregex Epoch 1 predictions correspond to peptides 85-94. Before Epoch 2, the training dataset was expanded to include the initial training set, the Epoch 1 predictions, the reversed sequence of EF hands 3 and EF hand 4, and additional EF hands (peptides 97-100 in Table S1). Thus, the Epoch 2 training dataset contained 100 peptides. POETmotif Epoch 2 predictions correspond to peptides 101–110, and POETregex Epoch 2 predictions correspond to peptides 111–120.

The column “POET r₁ ratio” reports the r₁-ratio value used for POET training or evaluation during the original workflow. This value was retained to preserve the exact dataset used by the algorithm. The corresponding SE represents the propagated standard error of the r₁ ratio calculated from the standard errors of the peptide and peptide-free GdCl₃ slope fits. When independent repeat measurements were available, the mean measured r₁ ratio, SD, and number of measurements are reported separately. These repeat measurements were used to assess experimental reproducibility but were not substituted retroactively into the POET training dataset.

The reversed EF hand sequences were measured to test whether sequence reversal could be treated as an additional dataset and thereby double the amount of available training data. However, MRI results showed that the regular and reversed sequences had different r_1_ ratios, indicating that the sequence order influenced the measured MRI contrast. Figure S3 provides a graphic representation of selected r_1_ ratio results from the initial dataset and POET predictions.

***Table S1. Complete list of peptides***

|  | Sample | Sequence | POET  r_1_ ratio | SE | Mean r_1_ ratio | SD | n |
| --- | --- | --- | --- | --- | --- | --- | --- |
| 1 | EF hand 1^3^ | DPDKDGTIDLKE | 1.51 | 0.03 | 1.60 | 0.14 | 3 |
| 2 | EF hand 2^3^ | DPDKDGTLDAKE | 1.52 | 0.02 | - | - | 1 |
| 3 | EF hand 3^3^ | DPDNDGTLDKKE | 1.86 | 0.05 | 1.85 | 0.02 | 3 |
| 4 | EF hand 4^3^ | NPDNDGTIDARE | 1.74 | 0.01 | 1.73 | 0.02 | 2 |
| 5 | Lanthanide binding^4^ | DKDGDTIDEREI | 1.31 | 0.03 | 1.35 | 0.07 | 2 |
| 6 | CaBM 2^5^ | DKDGDGTITTKE | 1.29 | 0.04 | 1.26 | 0.04 | 2 |
| 7 | CaBM^6^ | DKDGNGYISAAE | 0.93 | 0.03 | 0.91 | 0.02 | 2 |
| 8 | Training | DAPSFAKVVPNR | 0.39 | 0.07 | 0.62 | 0.30 | 4 |
| 9 | Training | KVNFNKAVSNLK | 0.40 | 0.09 | 0.63 | 0.31 | 4 |
| 10 | Training | LVKPMNKRWVRM | 0.40 | 0.11 | 0.68 | 0.27 | 5 |
| 11 | Training | LSNRRGREQYAG | 1.01 | 0.03 | - | - | 1 |
| 12 | Training | GIFKTTKCKHNS | 0.86 | 0.03 | - | - | 1 |
| 13 | Training | KVIRYVVAPMKL | 0.86 | 0.02 | - | - | 1 |
| 14 | Training | CHLKDLRKMGLR | 0.99 | 0.01 | - | - | 1 |
| 15 | Training | VKKYVTPGAPVY | 0.98 | 0.01 | - | - | 1 |
| 16 | Training | QPRMWVNVYTCY | 0.97 | 0.01 | - | - | 1 |
| 17 | Training | NQHTPPKPLNNA | 0.97 | 0.01 | - | - | 1 |
| 18 | Training | LHKDRRKPLPLP | 0.92 | 0.01 | - | - | 1 |
| 19 | Training | QLGVHRNPRWNQ | 1.01 | 0.01 | - | - | 1 |
| 20 | Training | HLRLNKNRMQKS | 1.01 | 0.01 | - | - | 1 |
| 21 | Training | NSSNHSNNMPCQ | 1.09 | 0.01 | 1.06 | 0.04 | 2 |
| 22 | Training | HQWHRHRPPIRR | 0.96 | 0.02 | - | - | 1 |
| 23 | Training | RWHHVNVLWQDR | 0.98 | 0.02 | - | - | 1 |
| 24 | Training | DNPHQLLPVNWR | 0.89 | 0.02 | - | - | 1 |
| 25 | Training | WFGLQRHLKKKD | 0.94 | 0.02 | - | - | 1 |
| 26 | Training | GQRWLYKMKDSM | 0.94 | 0.02 | - | - | 1 |
| 27 | Training | LDHTWGKWGHQS | 0.95 | 0.01 | - | - | 1 |
| 28 | Training | PVARKVVQICHP | 0.98 | 0.01 | - | - | 1 |
| 29 | Training | DPMRVRPWYVAR | 1.00 | 0.01 | - | - | 1 |
| 30 | Training | PGGVPFRLKMDP | 1.01 | 0.02 | - | - | 1 |
| 31 | Training | VCNRIEPLKPIL | 0.95 | 0.01 | - | - | 1 |
| 32 | Training | GNKKNWRWYKNR | 0.93 | 0.01 | - | - | 1 |
| 33 | Training | KPWHGCASRTKR | 0.93 | 0.01 | - | - | 1 |
| 34 | Training | NKAKACNGMKPR | 0.92 | 0.02 | - | - | 1 |
| 35 | Training | TIKPRMVKLPSV | 1.08 | 0.09 | 1.03 | 0.06 | 2 |
| 36 | Training | MKVAAAMAPKQV | 0.94 | 0.02 | - | - | 1 |
| 37 | Training | NWRDCLSLIVPN | 0.96 | 0.04 | - | - | 1 |
| 38 | Training | PLNIAISNSPDS | 0.51 | 0.03 | 0.81 | 0.22 | 4 |
| 39 | Training | DCLAVPPNAVTI | 0.95 | 0.03 | - | - | 1 |
| 40 | Training | VNSDPSNGQMRD | 1.22 | 0.04 | - | - | 1 |
| 41 | Training | RGKMPLRWMTRK | 0.96 | 0.01 | - | - | 1 |
| 42 | Training | IKGMNIKMPTDQ | 0.91 | 0.04 | - | - | 1 |
| 43 | Training | PEGVSWRVLKAN | 0.94 | 0.02 | - | - | 1 |
| 44 | Training | RPPMLNVVRVVG | 0.92 | 0.02 | - | - | 1 |
| 45 | Training | YEYEYEYEYEYE | 0.35 | 0.02 | 0.55 | 0.19 | 3 |
| 46 | Training | KSKSKSKSKSKS | 0.99 | 0.02 | - | - | 1 |
| 47 | Training | PKVVKDMPPWDT | 1.24 | 0.04 | - | - | 1 |
| 48 | Training | MLMKVPVIRRVW | 0.98 | 0.01 | - | - | 1 |
| 49 | Training | VARVDIDLQAIA | 0.94 | 0.01 | - | - | 1 |
| 50 | Training | VITNNWALAQVR | 0.96 | 0.01 | - | - | 1 |
| 51 | Training | TISDIVKVVIRS | 0.96 | 0.02 | - | - | 1 |
| 52 | Training | KSSKSSKSSKSS | 0.95 | 0.02 | - | - | 1 |
| 53 | Training | WLLDLRRQMDDT | 1.45 | 0.06 | - | - | 1 |
| 54 | Training | RDPDKNNRDWPI | 1.06 | 0.04 | - | - | 1 |
| 55 | Training | LDDDTPDRNRDW | 1.74 | 0.03 | - | - | 1 |
| 56 | Training | VGQNNDERRRQR | 1.01 | 0.01 | - | - | 1 |
| 57 | Training | PGPPAGPTLSNR | 1.02 | 0.01 | - | - | 1 |
| 58 | Training | NSPQLNLNPPQS | 0.99 | 0.01 | - | - | 1 |
| 59 | Training | NRSMPLPMNPGD | 1.16 | 0.03 | - | - | 1 |
| 60 | Training | NAARMNRLNDAM | 1.04 | 0.02 | - | - | 1 |
| 61 | Training | ANNNPWSGMMNG | 0.96 | 0.01 | - | - | 1 |
| 62 | Training | MWVKGMKHKKMK | 1.01 | 0.02 | - | - | 1 |
| 63 | Training | VPKDKRKKLTNP | 0.95 | 0.01 | - | - | 1 |
| 64 | Training | AACPNLAVAAPM | 0.94 | 0.02 | - | - | 1 |
| 65 | Training | VPKRLVVVMNTC | 1.06 | 0.01 | - | - | 1 |
| 66 | Training | EAPMPKVNVIVN | 1.05 | 0.01 | - | - | 1 |
| 67 | Training | PLPDNVVKAVVW | 1.02 | 0.01 | - | - | 1 |
| 68 | Training | CKPARTDWPPMP | 1.01 | 0.01 | - | - | 1 |
| 69 | Training | CATNAKNNNMRG | 1.00 | 0.01 | - | - | 1 |
| 70 | Training | VINKVISNPCVN | 0.99 | 0.01 | - | - | 1 |
| 71 | Training | LHSQWLKVDHLL | 1.01 | 0.01 | - | - | 1 |
| 72 | Training | QTATENSQMNSG | 1.14 | 0.02 | - | - | 1 |
| 73 | Training | KKKKKKKKKKKK | 1.01 | 0.01 | - | - | 1 |
| 74 | Training | KSSSKSSSKSSS | 0.96 | 0.01 | - | - | 1 |
| 75 | POET Motif (Epoch 1) | MDDLTGDNKNWV | 1.44 | 0.05 | - | - | 1 |
| 76 | POET Motif (Epoch 1) | QDTTMMTRLDTM | 1.06 | 0.02 | - | - | 1 |
| 77 | POET Motif (Epoch 1) | VDRNTDTDSVDT | 1.98 | 0.06 | - | - | 1 |
| 78 | POET Motif (Epoch 1) | KVDCDDVDRLDS | 2.00 | 0.04 | - | - | 1 |
| 79 | POET Motif (Epoch 1) | DMMHDKTDCNDT | 1.71 | 0.05 | - | - | 1 |
| 80 | POET Motif (Epoch 1) | TCTPTNTLNTMD | 1.23 | 0.04 | - | - | 1 |
| 81 | POET Motif (Epoch 1) | IDMTCSMTDDDT | 1.00 | 0.02 | - | - | 1 |
| 82 | POET Motif (Epoch 1) | QTCRNDGTIDKH | 1.10 | 0.01 | - | - | 1 |
| 83 | POET Motif (Epoch 1) | TNAGTMCTTNTD | 1.17 | 0.02 | - | - | 1 |
| 84 | POET Motif (Epoch 1) | CNDDTAGECTND | 2.08 | 0.02 | - | - | 1 |
| 85 | POET regex (Epoch 1) | CDVDPITCDNDD | 1.95 | 0.09 | - | - | 1 |
| 86 | POET regex (Epoch 1) | LDNPQEDADDDG | 2.12 | 0.04 | - | - | 1 |
| 87 | POET regex (Epoch 1) | LEDDIDCDNNDF | 1.77 | 0.03 | - | - | 1 |
| 88 | POET regex (Epoch 1) | DTMTLNDQETDD | 2.05 | 0.03 | - | - | 1 |
| 89 | POET regex (Epoch 1) | WHCDQTDDIDGH | 2.04 | 0.05 | - | - | 1 |
| 90 | POET regex (Epoch 1) | RDDETLDTLDSG | 2.05 | 0.05 | 2.08 | 0.03 | 2 |
| 91 | POET regex (Epoch 1) | NSAMADQADDDP | 1.12 | 0.02 | 1.03 | 0.08 | 3 |
| 92 | POET regex (Epoch 1) | IDCRGTEDDDPN | 2.30 | 0.04 | - | - | 1 |
| 93 | POET regex (Epoch 1) | RVDMDDTIDGMH | 1.83 | 0.08 | - | - | 1 |
| 94 | POET regex (Epoch 1) | NDGGDPMADNDN | 1.92 | 0.02 | - | - | 1 |
| 95 | EF hand 3 reversed^2^ | EKKDLTGDNDPD | 1.98 | 0.01 | - | - | 1 |
| 96 | EF hand 4 reversed^2^ | ERADITGDNDPN | 2.08 | 0.01 | - | - | 1 |
| 97 | Hans EF hand 1^7^ | NKDNDDSLEIAE | 1.62 | 0.03 | - | - | 1 |
| 98 | Hans EF hand 2^7^ | NPDGDTTLESGE | 1.64 | 0.02 | - | - | 1 |
| 99 | Hans EF hand 3^7^ | NKDGDQTLEMDE | 1.77 | 0.02 | - | - | 1 |
| 100 | Hans EF hans 4^7^ | DANKDGKLTAAE | 1.14 | 0.02 | - | - | 1 |
| 101 | POET Motif (Epoch 2) | DEDDDLECVDDA | 2.02 | 0.04 | - | - | 1 |
| 102 | POET Motif (Epoch 2) | GDLVDCDEDCDD | 1.76 | 0.04 | - | - | 1 |
| 103 | POET Motif (Epoch 2) | CDEDDEGVKDDD | 2.15 | 0.02 | - | - | 1 |
| 104 | POET Motif (Epoch 2) | NDGDGPCCEDDD | 2.07 | 0.01 | - | - | 1 |
| 105 | POET Motif (Epoch 2) | DDEDDTSGGDTK | 2.11 | 0.02 | - | - | 1 |
| 106 | POET Motif (Epoch 2) | GDDEDDQCHQDG | 2.17 | 0.01 | - | - | 1 |
| 107 | POET Motif (Epoch 2) | EDGDVIGSGKDD | 2.07 | 0.02 | - | - | 1 |
| 108 | POET Motif (Epoch 2) | TEGGTDDTGGND | 2.08 | 0.02 | - | - | 1 |
| 109 | POET Motif (Epoch 2) | CPTDDGHDDHDD | 2.10 | 0.03 | - | - | 1 |
| 110 | POET Motif (Epoch 2) | HDDGVGVDGGDG | 1.88 | 0.03 | - | - | 1 |
| 111 | POET regex (Epoch 2) | SDDNHSDLGDDL | 2.14 | 0.03 | - | - | 1 |
| 112 | POET regex (Epoch 2) | FEDHSDDTDEDT | 2.12 | 0.02 | - | - | 1 |
| 113 | POET regex (Epoch 2) | VDDSDPDDVKDT | 2.07 | 0.03 | - | - | 1 |
| 114 | POET regex (Epoch 2) | DDDSDEDSTDSH | 2.10 | 0.02 | - | - | 1 |
| *115* | POET regex (Epoch 2) | DSDEDTHVDCQE | 1.51 | 0.04 | - | - | 1 |
| *116* | POET regex (Epoch 2) | TQDSDDGMEDED | 2.16 | 0.02 | - | - | 1 |
| *117* | POET regex (Epoch 2) | STDTAGDDATEG | 2.09 | 0.03 | - | - | 1 |
| *118* | POET regex (Epoch 2) | ENHSGQHCHDTG | 1.05 | 0.02 | 1.02 | 0.05 | 2 |
| *119* | POET regex (Epoch 2) | VCEDHDPNDSHI | 1.81 | 0.04 | - | - | 1 |
| *120* | POET regex (Epoch 2) | DSDEDVHDTEWV | 1.88 | 0.06 | - | - | 1 |

**Figure S3**. r₁-ratio values for the initial dataset and POET-predicted peptides. GdCl₃ without peptide was used as the reference control, and higher r₁-ratio values indicate greater apparent MRI contrast under the screening conditions. Error bars represent the propagated standard error (SE) of the r₁ ratio calculated from the fitted slopes of the peptide–Gd and peptide-free GdCl₃ dilution curves. Orange bars indicate peptides with r₁-ratio values equal to or higher than EF3, the best-performing peptide in the initial dataset. The complete dataset is provided in Table S1.

Amino acid distribution

Figure S4 provides the complete 20-amino-acid percentage heatmap, including numerical values for each amino acid in the initial training dataset and the POET-predicted peptide groups.

**Figure S4.** Amino acid percentage heatmap corresponding to Figure 5B. Numerical values are shown in each cell to provide the full amino acid composition of the initial training dataset and the POET-predicted peptide groups.

**REFERENCES**

1.	Brautigam CA, Zhao H, Vargas C, Keller S, Schuck P. Integration and global analysis of isothermal titration calorimetry data for studying macromolecular interactions. *Nat Protoc.* 2016;11(5):882-894.

2.	Jeschke G. Electron Paramagnetic Resonance Spectroscopy. In: *Ullmann's Encyclopedia of Industrial Chemistry.*2013.

3.	Gutenthaler SM, Tsushima S, Steudtner R, et al. Lanmodulin peptides - unravelling the binding of the EF-Hand loop sequences stripped from the structural corset. *Inorg Chem Front.* 2022;9(16):4009-4021.

4.	Welch JT, Kearney WR, Franklin SJ. Lanthanide-binding helix-turn-helix peptides: Solution structure of a designed metallonuclease. *Proceedings of the National Academy of Sciences.* 2003;100(7):3725-3730.

5.	Sharma Y, Chandani S, Sukhaswami MB, Uma L, Balasubramanian D, Fairwell T. Modified helix-loop-helix motifs of calmodulin--The influence of the exchange of helical regions on calcium-binding affinity. *Eur J Biochem.* 1997;243(1-2):42-48.

6.	Wójcik J, Góral J, Pawłowski K, Bierzyński A. Isolated calcium-binding loops of EF-hand proteins can dimerize to form a native-like structure. *Biochemistry.* 1997;36(4):680-687.

7.	Mattocks JA, Jung JJ, Lin CY, et al. Enhanced rare-earth separation with a metal-sensitive lanmodulin dimer. *Nature.* 2023;618(7963):87-93.
